# Navigating the Gut-Cardiac Axis: Understanding Cardiovascular Complications in Inflammatory Bowel Disease

**DOI:** 10.7759/cureus.55268

**Published:** 2024-02-29

**Authors:** Tanya Sinha, Zukhruf Zain, Syed Faqeer Hussain Bokhari, Sarosh Waheed, Taufiqa Reza, Anthony Eze-Odurukwe, Mitwa Patel, Mohammed Khaleel I KH Almadhoun, Azlaan Hussain, Ibrahim Reyaz

**Affiliations:** 1 Medical Education, Tribhuvan University, Kirtipur, NPL; 2 Family Medicine, Aga Khan University Hospital, Karachi, PAK; 3 Surgery, King Edward Medical University, Lahore, PAK; 4 Medicine, Gujranwala Medical College, Gujranwala, PAK; 5 Medicine, Avalon University School of Medicine, Youngstown, USA; 6 Surgery, Salford Royal NHS Foundation Trust, Manchester, GBR; 7 Medicine, David Tvildiani Medical University, Tbilisi, GEO; 8 Medicine and Surgery, Mutah University, Karak, JOR; 9 Medicine and Surgery, Mayo Hospital, Lahore, PAK; 10 Internal Medicine, Christian Medical College and Hospital, Ludhiana, IND

**Keywords:** mesenteric ischemia, venous thromboembolism, peripheral artery disease, arrhythmias, stroke, heart failure, coronary artery disease, ibd, inflammatory bowel disease, cardiovascular complications

## Abstract

Inflammatory bowel disease (IBD) presents a complex interplay of chronic inflammation in the gastrointestinal tract and is associated with various extraintestinal manifestations, including cardiovascular complications (CVCs). IBD patients face an elevated risk of CVCs, including coronary artery disease, heart failure, arrhythmias, stroke, peripheral artery disease, venous thromboembolism, and mesenteric ischemia, necessitating comprehensive cardiovascular risk assessment and management. The intricate interplay between chronic inflammation, genetic predisposition, environmental factors, and immune dysregulation likely contributes to the development of CVCs in IBD patients. While the exact mechanisms linking IBD and CVCs remain speculative, potential pathways may involve shared inflammatory pathways, endothelial dysfunction, dysbiosis of the gut microbiome, and traditional cardiovascular risk factors exacerbated by the chronic inflammatory state. Moreover, IBD medications, particularly corticosteroids, may impact cardiovascular health by inducing hypertension, insulin resistance, and dyslipidemia, further amplifying the overall CVC risk. Lifestyle factors such as smoking, obesity, and dietary habits may also exacerbate cardiovascular risks in individuals with IBD. Lifestyle modifications, including smoking cessation, adoption of a heart-healthy diet, regular exercise, and optimization of traditional cardiovascular risk factors, play a fundamental role in mitigating CVC risk. Emerging preventive strategies targeting inflammation modulation and gut microbiome interventions hold promise for future interventions, although further research is warranted to elucidate their efficacy and safety profiles in the context of IBD. Continued interdisciplinary collaboration, advanced research methodologies, and innovative interventions are essential to address the growing burden of CVCs in individuals living with IBD and to improve their long-term cardiovascular outcomes.

## Introduction and background

Inflammatory bowel disease (IBD) is a chronic inflammatory condition of the gastrointestinal tract [1]. This inflammation leads to a variety of symptoms, such as abdominal pain, bloody diarrhea, urgency to defecate, fatigue, weight loss, malnutrition, fever, joint pain, and other extraintestinal manifestations [1,2]. IBD encompasses two main types: ulcerative colitis (UC) and Crohn's disease (CD) [1]. UC primarily affects the colon and rectum, causing inflammation and ulcers in the innermost lining of the large intestine. The inflammation typically starts in the rectum and progresses continuously upward [3]. On the other hand, CD can affect any part of the digestive tract, from the mouth to the anus, and involves inflammation through multiple layers of the intestinal wall [4]. The inflammation can be patchy, meaning there may be healthy areas interspersed with inflamed segments. It can also affect deeper layers of the bowel wall, potentially leading to complications such as strictures, fistulas, or abscesses [1]. While the exact cause of IBD remains unknown, it is believed to involve a combination of genetic, environmental, and immune system factors. These factors contribute to an abnormal immune response in the gut, leading to chronic inflammation and tissue damage [1-5].

Over the past few decades, there has been a concerning rise in the prevalence of IBD globally. According to Wang et al., estimates suggest that the number of individuals living with IBD worldwide has grown from 3.3 million in 1990 to 4.9 million in 2019 [6]. A study by Santiago et al. indicates a concerning incidence forecast until December 2023, emphasizing the increasing burden of the disease [7]. Over the period from 1990 to 2019, there has been a reduction in the total annual incidence by 12%, while deaths related to IBD surged by 172%, signaling a disproportionate increase in mortality compared to the decline in incidence [8]. A concerning prediction by Patel et al. suggests that age-standardized mortality rates due to IBD will rise by 0.9 per 100,000 individuals by the year 2040 [8]. Traditionally considered a disease of Westernized nations, IBD is now increasing significantly in previously low-incidence regions like Asia, Eastern Europe, and parts of Africa and South America [9].

Cardiovascular complications (CVCs) have emerged as a significant concern among individuals with IBD. The link between IBD and cardiovascular diseases is increasingly recognized, underscoring the importance of understanding this association. Numerous studies highlight that IBD patients may face a disrupted cardiovascular profile, leading to an elevated risk of cardiovascular complications such as coronary artery disease (CAD), stroke, thromboembolic events, pericarditis, myocarditis, arrhythmias, and venous and arterial thromboembolism [10-12]. According to a systematic review by Jaiswal et al., myocardial infarction (MI) occurred in 1.47% of IBD patients, while in UC and CD patients, it occurred at the rates of 30.96% and 34.14%, respectively. Cardiovascular disease events were observed in 1.95% of IBD patients [13]. Incidences of heart failure were recorded at 5.49% of IBD patients. Stroke events were noted at 0.95% for IBD patients, while in UC and CD patients, it occurred at the rates of 2.63% and 2.41%, respectively [13]. It has also been demonstrated that women particularly face an elevated risk of adverse cardiovascular outcomes among IBD patients [13,14].

This narrative review aims to provide a comprehensive overview of CVCs in IBD. It explores the rising prevalence of CVCs in IBD patients, delves into the potential mechanisms linking these conditions, and examines the various types of CVCs affecting this population. Additionally, this review discusses risk factors, modifiable aspects, and current strategies for managing and preventing CVCs in IBD patients. By synthesizing existing research and highlighting areas for further investigation, this review aims to contribute to a better understanding and improved cardiovascular health outcomes for individuals living with IBD. The scope of this review primarily focuses on published peer-reviewed literature on the topic of CVCs in IBD. It encompasses studies exploring risk factors, mechanisms, types of CVCs, and current management and prevention strategies. While acknowledging the potential role of specific IBD medications and nutritional interventions, the primary focus is on established knowledge and recognized associations between IBD and CVCs.

## Review

CVCs in IBD

IBD patients face an increased risk of CVCs, notably CAD. This heightened risk encompasses both UC and CD patients, with studies consistently showing a higher risk compared to the general population [13,15,16]. Notably, IBD patients also exhibit an increased risk of angina, MI, and HF stemming from CAD, highlighting the systemic impact of chronic inflammation beyond the gut [12,17]. Studies show that CD has a higher association with CAD and MI as compared to UC [18-20]. The risk of HF in IBD is influenced by factors such as disease type, disease activity, and disease severity, with severe IBD potentially posing a higher risk. Chronic inflammation, increased myocardial damage, and nutritional deficiencies contribute to the elevated risk of HF in IBD patients. Arrhythmias, although less common, also present a significant concern in IBD patients, with studies suggesting an increased risk compared to the general population [21-23]. Possible causes include chronic inflammation, electrolyte imbalances, autoimmune mechanisms, and medication side effects. Types of arrhythmias observed in IBD patients include atrial fibrillation/flutter, supraventricular tachycardia, and ventricular arrhythmias [21,22]. These risks increase with IBD flares and persistent disease.

Other CVCs associated with IBD include stroke, peripheral artery disease (PAD), and venous thromboembolism (VTE) [24,25]. IBD patients face an elevated risk of stroke compared to the general population, with similar mechanisms implicated as in CAD [26]. Limited research exists on the association between IBD and PAD, but emerging evidence suggests a potential link. Additionally, IBD patients exhibit an increased risk of VTE, including deep vein thrombosis (DVT) and pulmonary embolism (PE), attributed to factors like inflammation, hospitalization, and medication use [27-29]. Lastly, while less common, mesenteric ischemia poses a unique challenge in IBD patients. The chronic inflammation and vascular dysfunction characteristic of IBD increases the risk of mesenteric ischemia, necessitating early recognition and proactive management to prevent severe consequences such as bowel perforation [30]. In summary, IBD patients face a spectrum of CVCs, including CAD, HF, arrhythmias, stroke, PAD, VTE, and mesenteric ischemia, emphasizing the importance of comprehensive cardiovascular risk assessment and management in this population.

Potential mechanisms linking IBD and CVCs

The growing concern surrounding elevated CVCs in patients with IBD stems from the recognition of shared inflammatory pathways underlying both conditions. While the intricate interactions within these pathways involve numerous factors, certain key players emerge as central to the pathogenesis of both IBD and atherosclerosis. Pro-inflammatory signaling molecules, including interleukin-1β (IL-1β), tumor necrosis factor-α (TNF-α), and interleukin-6 (IL-6), are notably elevated in both IBD and atherosclerosis [31-33]. These molecules orchestrate chronic inflammation, endothelial dysfunction, and smooth muscle cell proliferation, thereby contributing to plaque formation and vascular damage. In essence, the perpetuation of systemic inflammation fueled by these cytokines acts as a common driving force behind the development and progression of both IBD and CVCs. The endothelium, a crucial regulator of vascular function, plays a pivotal role in maintaining cardiovascular health. In healthy individuals, the endothelium promotes vasodilation, inhibits thrombosis, and regulates inflammation [34,35]. However, endothelial dysfunction disrupts this delicate balance. Decreased nitric oxide (NO) bioavailability, a consequence of endothelial dysfunction in IBD, leads to vasoconstriction and impaired vasodilation. This compromised vascular reactivity contributes to the pathogenesis of atherosclerosis, hypertension, thrombosis, MI, stroke, and other related CVCs in patients with IBD [36-38].

C-reactive protein (CRP), a well-established marker of systemic inflammation, is frequently utilized to assess disease activity in IBD [39]. However, emerging evidence suggests that elevated CRP levels not only reflect ongoing inflammation but also serve as predictors of future cardiovascular events in both IBD and the general population. Elevated CRP levels contribute to atherogenesis by promoting platelet aggregation and impairing vascular dilation, thereby exacerbating cardiovascular risk [12,40,41]. Another factor implicated in the increased cardiovascular risk observed in IBD patients is homocysteine. Elevated levels of homocysteine have been linked to an augmented risk of cardiovascular events. Studies suggest that IBD patients may exhibit elevated homocysteine levels due to impaired absorption and reduced levels of vitamin B12 [42,43]. Elevated homocysteine levels can damage blood vessel walls and promote thrombosis, thereby further augmenting the risk of CVCs in IBD. As studies delve deeper into understanding the intricate relationship between IBD and the heightened risk of CVCs, emerging evidence points towards the potential role of gut microbiota dysbiosis as a significant contributing factor. IBD is characterized by an imbalance in the gut microbiome, marked by a decrease in beneficial bacteria and an increase in potentially harmful microbes [44,45].

This dysbiosis sets the stage for chronic inflammation within the gut, culminating in local tissue damage, heightened gut permeability, and the release of pro-inflammatory cytokines. Consequently, bacterial products such as lipopolysaccharides (LPS) and cytokines translocate into the bloodstream, initiating immune responses and fostering systemic inflammation [46]. Systemic inflammation in IBD is intricately linked to the development of endothelial dysfunction and the progression of atherosclerosis. Moreover, dysbiosis-induced metabolic alterations, notably the increased production of trimethylamine-N-oxide (TMAO), have been implicated in elevating cardiovascular risk. TMAO promotes the formation of foam cells, endothelial dysfunction, and platelet activation, thereby contributing to plaque formation and thrombosis [46,47]. Conversely, the normal gut flora produces beneficial short-chain fatty acids (SCFAs) such as butyrate and propionate, which exhibit anti-inflammatory properties and may confer protection against cardiovascular disease [48]. However, dysbiosis in IBD disrupts this equilibrium, leading to reduced SCFA production and further compromising vascular health. Additionally, dysbiosis-induced alterations in bile acid metabolism impact lipid absorption and metabolism, thereby exerting additional influence on cardiovascular risk [49,50].

While the evidence implicating gut microbiota dysbiosis in IBD-related atherogenesis is promising, further research is imperative to establish more definitive causal relationships and identify specific microbial targets for intervention. Investigations exploring the therapeutic potential of prebiotics, probiotics, and fecal microbiota transplantation (FMT) hold promise in modulating the gut microbiome and potentially mitigating the risk of CVCs in IBD patients [51-53]. The interplay between gut microbiota and cardiovascular health in the context of IBD underscores the multifaceted nature of the disease and highlights the importance of holistic approaches to patient management. Integrating strategies aimed at restoring gut microbial balance alongside conventional therapies may offer novel avenues for reducing cardiovascular risk in individuals with IBD. Moreover, elucidating the mechanisms underlying gut microbiota dysbiosis and its impact on cardiovascular health could pave the way for personalized interventions tailored to individual patient profiles.

In addition to the previously discussed mechanisms, thrombosis and malnutrition emerge as significant contributors to the heightened risk of CVCs in patients with IBD. The chronic inflammation characteristic of IBD disrupts the delicate balance between pro-thrombotic and anti-thrombotic factors, leading to a hypercoagulable state and promoting thrombus formation [54]. This dysregulation exacerbates the risk of thrombosis, particularly in IBD patients who may already be predisposed to such events due to factors like dehydration and malnutrition commonly seen in this population. Dehydration and malnutrition, prevalent issues among individuals with IBD, further compound the risk of thrombosis. Deficiencies in essential vitamins and minerals such as folate, vitamin B12, and iron can contribute to various cardiovascular risk factors, including elevated homocysteine levels, iron deficiency anemia, and dyslipidemia [55-57]. These nutritional deficiencies not only exacerbate the pro-thrombotic state but also contribute to the overall burden of cardiovascular risk factors in IBD patients. Moreover, the risk of CVCs varies throughout the course of IBD. It is highest during active disease flares when inflammation levels are elevated and decreases during periods of remission. Additionally, the duration of IBD illness plays a significant role in the cumulative risk of developing atherosclerosis and CVCs. As individuals live with IBD for longer periods, the chronic inflammation and associated cardiovascular risk factors exert a more pronounced effect, further amplifying the likelihood of cardiovascular events [15].

Risk factors and modifiable aspects

The presence of traditional cardiovascular risk factors such as smoking, hypertension, dyslipidemia, diabetes, and obesity in patients with IBD significantly compounds the overall cardiovascular risk [15]. Among these factors, smoking stands out as one of the most crucial modifiable risk factors in the management of both IBD and associated CVCs. Not only does smoking exacerbate symptoms of IBD, but it also heightens the risk of atherosclerosis, MI, and stroke. Implementing support programs and cessation aids can prove instrumental in controlling this detrimental risk factor [58].

Endothelial dysfunction, as discussed earlier, is a hallmark of IBD and contributes to hypertension. Furthermore, certain medications utilized in the management of IBD, such as corticosteroids, may exacerbate hypertension by elevating blood pressure levels [59]. Whether as a consequence of IBD or as an independent comorbidity, hypertension amplifies the overall cardiovascular risk, particularly HF, by imposing additional strain on the heart [13]. Therefore, strict control of blood pressure through lifestyle modifications and appropriate medication, including antihypertensive drugs, is imperative in the management of IBD.

Dyslipidemia, characterized by elevated levels of low-density lipoprotein (LDL) cholesterol and triglycerides, poses an increased risk of atherosclerosis and cardiovascular events. Patients with IBD are particularly susceptible to dyslipidemia due to chronic inflammation and malnutrition [60]. Weight fluctuations are common among individuals with IBD, resulting from disease activity, medication side effects, or dietary changes. Obesity, a well-established cardiovascular risk factor, further exacerbates the cardiovascular risk in IBD patients. Additionally, weight gain may occur during the treatment of IBD, further elevating the risk of cardiovascular events [61]. It is therefore essential to optimize the lipid profile and maintain a safe basal metabolic index (BMI) through dietary interventions, regular exercise, and potentially the use of lipid-lowering medications such as statins.

IBD medications, particularly corticosteroids, can also impact cardiovascular health. Corticosteroids may induce hypertension, insulin resistance, and dyslipidemia, thereby exacerbating traditional cardiovascular risk factors [13,62]. Consequently, the cardiovascular risks associated with corticosteroid use must be carefully weighed against the benefits of managing inflammation in IBD. In summary, a comprehensive approach addressing traditional cardiovascular risk factors alongside the management of IBD is essential in mitigating the overall cardiovascular risk burden in affected individuals.

Management and prevention strategies

While the identification of CVCs in IBD patients is crucial, effectively managing their cardiovascular risk poses significant challenges. The multifactorial nature of IBD, coupled wSCORE2ith its systemic inflammatory effects, complicates the management of traditional cardiovascular risk factors. Moreover, the impact of IBD medications, particularly corticosteroids, on cardiovascular health necessitates a nuanced approach to treatment. Early identification of CVCs in IBD patients is crucial due to their heightened risk compared to the general population. Proactive screening for traditional cardiovascular risk factors, including blood pressure, cholesterol, LDL, blood glucose levels, and CRP, enables timely intervention [63]. Risk calculation tools (such as SCORE2 and QRISK3) can be used to estimate individual CVD risk based on demographics, clinical history, and laboratory parameters. This allows for risk stratification, helping categorize patients into low, moderate, or high-risk groups, leading to tailored management strategies [64].

One of the primary challenges lies in addressing the interplay between chronic inflammation and traditional cardiovascular risk factors. While inflammation plays a central role in the pathogenesis of both IBD and CVCs, traditional risk factors such as smoking, hypertension, dyslipidemia, and obesity further exacerbate cardiovascular risk in these patients. Balancing the management of inflammation with the optimization of traditional risk factors requires a multidisciplinary approach involving gastroenterologists, cardiologists, endocrinologists, and nutritionists. This multidisciplinary approach facilitates early identification of CVCs, risk assessment, and the implementation of appropriate interventions. Shared decision-making involving the patient ensures the optimization of outcomes while balancing the management of IBD and cardiovascular risk reduction strategies.

Nutritional support and specific dietary interventions tailored to the needs of IBD patients can help manage inflammation and improve cardiovascular health. Incorporating omega-3 fatty acids, probiotics, and prebiotics into the diet may have anti-inflammatory effects and support gut health. Additionally, avoiding trigger foods and potential allergens can help minimize inflammation and promote overall well-being [65-67]. Modulating the gut microbiome through dietary interventions, probiotics, or fecal microbiota transplantation may emerge as novel strategies for reducing inflammation and cardiovascular risk in IBD patients. In conclusion, addressing traditional cardiovascular risk factors, understanding the impact of IBD medications, promoting lifestyle modifications, and considering nutritional support are essential in managing cardiovascular health in IBD patients. A multidisciplinary approach involving healthcare providers, dietitians, and lifestyle coaches is necessary to optimize cardiovascular outcomes in this patient population.

Future directions and challenges

Significant progress has been made in understanding the link between IBD and CVCs; however, several limitations and unanswered questions remain. Addressing these knowledge gaps through dedicated research is crucial for improving patient outcomes and developing effective preventative strategies. The exact underlying mechanisms leading to cardiovascular events in IBD remain poorly understood. More research is required to elucidate the complex interplay between inflammation, immune dysregulation, and CVD pathogenesis in IBD. IBD encompasses a spectrum of diseases with varying clinical phenotypes and courses. Most research focuses on IBD as a whole, neglecting potential differences in CVC risk between UC and CD or across different disease severities. More studies are needed to understand these nuances and guide targeted interventions. Existing studies primarily rely on observational data, making it difficult to establish definite causal relationships between specific factors and CVC development. Larger, prospective studies with robust methodologies are needed to provide stronger evidence. Longitudinal cohorts with comprehensive clinical and cardiovascular assessments can provide valuable insights into disease trajectories and risk factors. While lifestyle modifications and traditional CVD management are crucial, their effectiveness in specifically mitigating CVC risk in IBD patients might be suboptimal. Novel therapeutic approaches tailored to the unique pathophysiology of IBD-CVC should be explored.

Targeted immunomodulatory therapies and microbiome-based interventions hold promise for mitigating the risk of CVCs in IBD. This includes studying drugs targeting specific inflammatory pathways linked to both IBD and CVD, like IL-17 or Janus kinase (JAK) inhibitors, and exploring immunomodulators with additional cardioprotective properties [68-70]. However, the long-term cardiovascular safety profile of these agents requires further investigation. Dietary or fecal microbiota transplant strategies to manipulate the microbiome for prevention are a promising avenue to target dysbiosis-associated CVC risk in IBD [51-53]. Genetic markers associated with increased CVC risk in specific IBD subsets should be explored for personalized preventative measures. Advanced imaging techniques and tools like PET scans or coronary artery calcium scoring can be utilized to improve the early detection and risk stratification of CVCs in IBD. Machine learning and artificial intelligence can be employed for analyzing large datasets to identify new risk factors, predict disease progression, and personalize treatment strategies [71,72]. Fostering collaboration between gastroenterologists, cardiologists, immunologists, and other specialists can result in a comprehensive understanding of the IBD-CVC link. By addressing current limitations and actively pursuing promising avenues, researchers can pave the way for more effective strategies to prevent and manage these life-threatening complications, ultimately improving the well-being of patients with IBD.

## Conclusions

IBD poses a significant burden on affected individuals, with a rising global prevalence and associated mortality rates. The recognition of CVCs as a major concern among IBD patients underscores the need for a comprehensive understanding and management of these conditions. Elevated risks of CAD, stroke, HF, arrhythmias, and thromboembolic events are observed in IBD patients, necessitating proactive screening and risk assessment. The intricate interplay between chronic inflammation, traditional cardiovascular risk factors, medication effects, and disease severity contributes to the development of CVCs in IBD. Collaborative care involving gastroenterologists and cardiologists is crucial for early identification and tailored management strategies. Lifestyle modifications, including smoking cessation, a healthy diet, and regular exercise, are fundamental to mitigating CVC risk. Moreover, emerging preventive strategies targeting inflammation modulation and gut microbiome interventions hold promise for future interventions. Despite advancements, challenges remain in understanding the mechanistic links between IBD and CVCs, highlighting the need for further research and personalized therapeutic approaches.
